# Rat and fish peripheral blood leukocytes respond distinctively to *Anisakis pegreffii* (Nematoda, Anisakidae) crude extract

**DOI:** 10.3389/fcimb.2022.1042679

**Published:** 2022-12-15

**Authors:** Jerko Hrabar, Mirela Petrić, Serena Cavallero, Marco Salvemini, Stefano D’Amelio, Ivona Mladineo

**Affiliations:** ^1^ Laboratory of Aquaculture, Institute of Oceanography and Fisheries, Split, Croatia; ^2^ University Department of Marine Studies, University of Split, Split, Croatia; ^3^ Department of Public Health and Infectious Diseases, University of Rome, Sapienza, Rome, Italy; ^4^ Department of Biology, University of Naples Federico II, Naples, Italy; ^5^ Laboratory of Functional Helminthology, Institute of Parasitology, Biology Centre of Czech Academy of Sciences, Ceske Budejovice, Czechia

**Keywords:** *Anisakis*, crude extract, fish, rat, peripheral blood leukocytes, RNA-Seq

## Abstract

Infective third-stage larvae (L3) of the marine nematode *Anisakis pegreffii* cause inflammation and clinical symptoms in humans, their accidental host, that subside and self-resolve in a couple of weeks after L3 die. To characterise the differences in an early immune response of a marine vs. terrestrial host, we stimulated peripheral blood leukocytes (PBLs) of fish (paratenic host) and rat (accidental, human-model host) with *A. pegreffii* crude extract and analysed PBL transcriptomes 1 and 12 h post-stimulation. Fish and rat PBLs differentially expressed 712 and 493 transcripts, respectively, between 1 and 12 h post-stimulation (false discovery rate, FDR <0.001, logFC >2). While there was a difference in the highest upregulated transcripts between two time-points, the same Gene Ontologies, biological processes (intracellular signal transduction, DNA-dependent transcription, and DNA-regulated regulation of transcription), and molecular functions (ATP and metal ion binding) were enriched in the two hosts, showing an incrementing dynamic between 1 and 12 h. This suggests that the two distinct hosts employ qualitatively different transcript cascades only to achieve the same effect, at least during an early innate immunity response. Activation of later immunity elements and/or a combination of other host’s intrinsic conditions may contribute to the death of L3 in the terrestrial host.

## Introduction

Phagocytes cannot easily damage parasitic helminths given their extensive size, but they can be efficiently activated by the parasite excretory/secretory products (ESPs). In higher vertebrates, dendritic cells (DCs) play a primal role in the recognition and capture of helminth ESPs through toll-like receptors (TLRs), C-type lectin receptors, and nucleotide-binding domain leucine-rich repeat-containing receptors, mediating the pro-inflammatory response through ESP processing and presentation to the T cells (Motran et al., 2018). However, those helminths that survive and reproduce in the human host have evolved different strategies to evade and modify host immune response, contributing to the development of a non-lethal infection (Inclan-Rico and Siracusa, 2018).

Helminths can deviate particular elements of the host inflammatory network and express both common and host–parasite system-specific products to achieve the infection. It seems that the sooner in the process the silencing begins, the better are the chances for efficient parasite colonisation of the host. For example, the murine nematode *Heligmosomoides polygyrus* produces HpARI (*H. polygyrus* alarmin release inhibitor) already during parasite migration and tissue destruction. This ESP blocks elements of the host damage-associated molecular patterns such as IL-33, one of the host’s alarmins produced during epithelial necrosis (Osbourn et al., 2017). In contrast, *Schistosoma mansoni* evolved different antigens recognised by TLR2 and TLR4 that unexpectedly silence the expression of IL-12 in DCs, promoting the polarisation toward a Th2-type response. While TLR2 and TLR4 stimulated by bacterial LPS usually prompt Th1 response through activation of the mitogen-activated MAP kinases (MAPK), p38, JNK (c-Jun N-terminal kinase), and ERK (extracellular signal-regulated kinase), *S. mansoni* and filarial antigens suppress p38 and JNK and inhibit the production of IL-12 in an ERK-dependent manner. The molecular mechanism is still not clear and may depend on the association of the TLRs with different co-receptors, especially the carbohydrate type, which may interfere with the downstream signalling cascade (Gantner et al., 2003). Another specificity of the helminth-driven Th2 is the lack of an early IL-4 production, compensated by the interaction of tissue-derived factors, such as the alarmins, thymic stromal lymphopoietin (TSLP), matrix metalloproteinase 2 (MMP-2), IL-33, IL-25, and eosinophil-derived neurotoxin (EDN) on DCs (Motran et al., 2017). Afterward, basophils and eosinophils sense the pathogen-associated molecular patterns (PAMPs), start processing and presenting helminth antigens to the naïve T cells, and then finally produce IL-4 and manage the development and amplification of the Th2 response (Makepeace et al., 2012).

Even in the well-established host–parasite interactions (e.g., schistosomes and humans), there is still a lot to elucidate on interacting immune ligands and receptors. For parasites that switch hosts through purely accidental events (e.g., ingestion through the food chain), the success of new niche colonisation is largely dependent on interaction with the “unknown” immune system. Interestingly, these accidental host switches account more for the expanding of the parasite range than the process of co-speciation itself, suggesting them as a valuable model to inspect closer the onset of host–parasite Red Queen effect (Martens and Schön, 2000).

Herein, we used *Anisakis pegreffii* crude extract to assess pathways expressed by fish (evolutionary-established paratenic host model) and rat (evolutionary un-established accidental host model) peripheral blood leukocytes (PBLs) at the onset of immune reaction, assuming a bias toward the Th1/Th2 response conditioned by the host type. Anisakids colonised the marine environment approximately 360.47 Mya (Early Carboniferous) (Li et al., 2018) and therefore fail to survive and develop in humans. They are however able to temporarily infect humans during their larval stage through the ingestion of raw or undercooked infected fish. The infection can elicit four main illness types, gastric, intestinal, ectopic, and gastro-allergic (Nieuwenhuizen, 2016), expressing mostly mild but sometimes severe symptomatology (Baptista-Fernandes et al., 2017). It is self-limiting because the infective third-stage larvae (L3) are unable to develop into reproductively active adults, which conditions their death within human tissues and decay. The total crude antigens from dead parasite further promote a localised eosinophilic granulomatosis and/or hypersensitisation to the *Anisakis* antigens. We hypothesise that the terrestrial mammals have become “evolutionarily resilient” to modulation of their immune response by the nematode. This can sometimes result in detrimental consequences, such as in the case of anaphylactic reaction in hypersensitised individuals. In the accidental host (e.g., rat), a strong immune reaction to L3 is characterised by a marked expression of specific proinflammatory cytokines and alarmins (calprotectins S100A8/S100A9). These are regulated *via* the expression of leukocyte-silencing miRNA (miRNA-451 and miRNA-223) (Bušelić et al., 2018; Hrabar et al., 2019). Conversely, in fish experimentally infected by *Anisakis*, IgM and CD8 are downregulated and cytokines (i.e., IL-1β, IL-4/IL-13, IL-6, IL-8, IL-10, IL-22, TNFα, and TGFβ) are not perturbated at all. This suggests that larval ESPs contribute to the immune silencing in this host (Haarder et al., 2013).

To compare the early response to *Anisakis pegreffii* total antigens (*Anisakis* crude extract; ACE), we stimulated the naïve peripheral blood leukocytes (PBLs) of the rat *Rattus norvegicus* and the seabass *Dicentrarchus labrax* representing accidental and natural hosts, respectively, evaluated transcriptomic profiles (RNA-seq) of the *Anisakis*-treated PBLs over time (1 and 12 h), and measured target gene expression (qPCR) in sensitised vs. non-sensitised rat spleen.

## Materials and methods

### ACE

Parasite crude extract was used to mimic the release of somatic antigens from the decaying L3 that fail to survive in the accidental host, such as terrestrial mammals, simulating the scenario in the pathogenesis of the anisakiasis (Trumbić et al., 2021). Type I *Anisakis* larvae (later molecularly identified as *A. pegreffii*) were collected from the blue whiting *Micromesistius poutassou*, freshly caught in the C1 fishing zone of the Adriatic Sea (FAO 37.2.1), provided by a trusted dealer. Briefly, actively moving larvae were washed several times in physiological saline solution and checked under an Olympus BX 40 light microscope (Olympus Corp., Shinjuku, Tokyo, Japan) to confirm the type I larvae identity. Approximately 400 larvae were washed in PBS (pH = 7.4) and deep frozen for 15 min at -80°C. Larvae were thawed, dried on paper, weighted to obtain 0.25 g of larvae/1 ml PBS, frozen again, and manually homogenised. Afterward, the homogenate was sonicated on ice for 60 s (10% duty cycles, 20% power) and centrifuged (600 g/10 s, at 4°C). The supernatant was collected and filtered through 0.45-mm filters. The concentration was calculated according to Bradford using standard dilutions of bovine serum albumin, resulting in 4 mg/ml of ACE from approximately 400 larvae. Molecular identification of a larval subsample taken before the crude extract preparation was done by PCR-based restriction fragment length polymorphism (PCR-RFLP) as described before (Bušelić et al., 2018), and the species identity of *A. pegreffii* was confirmed. While not all L3 used in experiment were molecularly identified, given that only *A. pegreffii* is distributed in the Adriatic, it is substantiated to claim that all L3 belong to this species.

### PBL stimulation with ACE

The sample size was determined following the guidelines by Genser et al. (2007). Nine European seabass (*Dicentrarchus labrax*) were anesthetised in a tank containing tricaine methane-sulfonate (MS-222, 0.2 mg/l), and blood samples (0.4–0.5 ml) were collected with 1-ml syringes from the caudal vein in EDTA-coated tubes and kept on ice.

Five rats (*Rattus norvegicus*) (476 ± 112.4 g) were anesthetised using a mixture of anaesthetic and analgesic (xylazine 5–10 mg/kg and Ketamidor 50-100 mg/kg, intra-peritoneal injection), and blood samples (0.2–0.3 ml) were collected with a 23G needle attached to a syringe from the tail vein in the lithium heparin-coated tubes and kept on ice.

Each animal species originated from a single parentage, representing the full siblings. Blood samples collected from the seabass and rats were diluted (1:5 ration) with incomplete medium (Leibovitz’s L-15 medium, Life Technologies), supplemented with 10 units/ml of heparin, 2% foetal calf serum (FCS) (Invitrogen), 100 U/ml penicillin, and 100 µg/ml streptomycin (P/S) (all Sigma-Aldrich). PBLs were isolated from the whole blood by centrifugation at 2,000 g for 20 min using 51% iso-osmotic Percoll (Gibco) solution and subsequently washed twice in incomplete medium. To ensure the number and viability of PBLs greater than 95%, trypan blue staining was used for the enumeration of dead cells in a haemocytometer. Cells were subsequently resuspended and diluted in the complete medium (L-15 supplemented with 15% FCS and P/S) to a final concentration of 10^7^ cells/ml, seeded in six-well plates, and left for acclimatisation for 3 h at room temperature. Cultures were then stimulated with 10 µg/ml of ACE and incubated in the air atmosphere at 37°C and 20°C (rat and fish PBLs, respectively). In the control PBLs, only culture media were added. The treatments were terminated after 1 and 12 h by resuspending cells in 1 ml of TRI Reagent (Ambion).

In total, eight samples were sequenced for each of the PBL experiments (rats and seabass): three samples after 1 h of stimulation, three samples after 12 h of stimulation, and one sample of non-stimulated cells after 1 h and one sample of non-stimulated cells after 12 h. Each sample consisted of two technical replicates. Fish samples collected at 1 h were compared with samples collected at 12 h, and rat samples collected at 1 h were compared with rat samples collected at 12 h. Samples of unstimulated cells at 1 and 12 h were used to check the sequencing quality and were not used for downstream statistical analyses.

### cDNA preparation and Illumina next-generation sequencing

Total RNA was isolated, and cDNA was prepared as described in Bušelić et al. (2018). Illumina NextSeq 500 (length of the reads 150 bp) was used for paired-end sequencing of a total of eight pooled PBL samples prepared from test and control treatments.

### Bioinformatic analyses of sequence data

The quality assessment of the raw reads derived from different lanes done using FASTQC (Andrews, 2014) evidenced no lane effects and good-quality reads, which were therefore joined into two paired FASTQ files per sample. Two samples did not meet criteria and were discarded from the downstream analyses (RA1h_3 and FA12h_3). Illumina adapters were trimmed choosing clipping by sliding window (quality threshold 15, window size 4), and reads shorter than 50 bases were removed using Trimmomatic (Bolger et al., 2014). The low-complexity reads (entropy threshold 70) and reads with more than 10% ambiguous (N) nucleotides were removed using PRINSEQ (Schmieder and Edwards, 2011).

The trimmed filtered reads for each species were used to perform a *de novo* assembly using the Trinity software (Haas et al., 2013). The quality check for assembled transcriptome completeness were inferred with BUSCO (Simão et al., 2015). Reads were then aligned to the corresponding *de novo* assembled transcriptomes using Bowtie, and their relative expression was calculated using the RSEM package filtering out the lowly expressed transcripts (fragments per kilobase million, FPKM <1 cutoff value). After cross-sample normalisation, differential expression analysis of transcripts (DEGs) in ACE-treated PBLs over time (1 and 12 h) was carried out using the edgeR package in R using logFC >2 and FDR <0.05 parameters (false discovery rate) (Robinson et al., 2010; McCarthy et al., 2012). Comparisons for DEGs were performed between the two times of exposure of fish and rat PBL to ACE (1 h vs. 12 h). Upregulated and enriched transcripts in two hosts were identified according to the two conditions of interest (i.e., 1 vs. 12 h), meaning that a transcript can be only downregulated or upregulated in 1 or 12 h, not at both times.

Transcripts annotations were obtained using Annocript (Musacchia et al., 2015) taking into account coding regions identified in each transcriptome using the BLASTn, tBLASTn, and rpsBLAST algorithms. Gene Ontology (GO) classifications according to the biological process (BP), molecular function (MF), and cellular component (CC) categories were obtained by querying the SwissProt, UniRef, and TrEMBL databases. GO and pfam (protein families) enrichment analyses were performed, and transcripts with GO terms relative to ribosomal and nucleolar stress were further analysed, with the aim to evaluate their putative role in host-response pathways.

### Target-specific qPCR in *Anisakis*-sensitised rats

Five rats (535 ± 29.5 g) were injected with 0.5 ml of ACE (0.5 µg ACE/ml of PBS, prepared 1:1 in complete Freund’s adjuvant) at the tail base, under isoflurane anaesthesia. On the fifth day p.i., rats were sacrificed and pieces of spleen were stored in RNAlater. An additional five *Anisakis*-unsensitised rats were injected with the PBS and complete Freund’s adjuvant (1:1) then sacrificed to obtain spleens as controls. Subsequently, RNA was isolated with TRI Reagent (Ambion) as per manufacturer’s protocol and its quality/quantity checked. cDNA was synthesised from 1 µg of total RNA using PrimeScript 1st Strand cDNA Synthesis Kit (Takara). Before reverse transcription, RNA was treated with DNase I (Thermo Scientific) to remove residual genomic DNA. To evaluate the systemic immune response of animals exposed to *A. pegreffii* crude extract, the expression of several immunity genes was measured by qPCR (**Table 1**). Cycling conditions were set according to Hrabar et al. (2019), except that cDNA was diluted 1:10. The Primer3 (v. 4.1.0.) web interface was used to design specific primer sets (**Table 1**). Efficiency of each primer pair, where E = 10^(− 1/slope)^, was calculated from sixfold serial dilutions of equal mole amounts of purified PCR products obtained in the PCR reaction using the respective primer pair. The efficiency of all runs was checked to be always higher than 90%, and the specificity was verified by analysis of melting curves.

**Table 1:** Immunity-related genes with designated primer sets used to evaluate the response in *Anisakis*-sensitised rats measured by qPCR.

	Primer name	Nucleotide sequence (5′ → 3′)	Product size (bp)	Efficiency (%)
Interleukin-1β(*Il-1β*)	IL1β_F IL1β_R	TCAAGCAGAGCACAGACCTG ACTGCCCATTCTCGACAAGG	155	94.05
Interleukin-6(*Il6*)	IL6_F IL6_R	TCTCCGCAAGAGACTTCCAGC TGGTCTGTTGTGGGTGGTATCC	122	92.88
Interleukin-18(*Il18*)	IL18_F IL18_R	AGGACTGGCTGTGACCCTAT TCCTGGCACACGTTTCTGAA	151	94.7
Tumor necrosis factor alpha(*Tnf-α*)	TNF_F TNF_R	GCCACCACGCTCTTCTGTCTA GGTTTGCTACGACGTGGGCT	166	94.32
Chemokine (C-C motif) ligand 3(*Ccl3*)	CCL3_F CCL3_R	CACCCTCTGTTACCTGCTCA ATCTGCCGGTTTCTCTTGGT	240	96.6
Intracellular adhesion molecule-1(*Icam-1*)	ICAM1_F ICAM1_R	CGGTGCTCAGGTATCCATCC CTGTCTTCCCCAATGTCGCT	189	90.61
**Housekeeping genes**				
Vacuolar protein sorting-associated protein 29(Vps29)	VPS29_F VPS29_R	TGGTGACTGAACGGAATCCC GGACATCACCAGCCAGAGTC	210	90.26
Glucose-6-phosphate isomerase (*Gpi*)	GPI_F GPI_R	GACGTGATGCCAGAGGTCAA GTTTTGGCAATGTGGGTCCC	236	85.47

Log2 transformation and differential expression analysis of target genes were performed in R (ver. 4.1.0) (R Core Team, 2020). Normalisation of the target gene expression was done on an individual basis against the geometric mean of the two selected housekeeping genes (*vps29*, *gpi*). Fold changes were calculated relative to the control as a group. Fold change greater than 2 (i.e., log2FC ≥1) was considered biologically significant, i.e., a change in gene expression that could result in enough protein secretion to manifest a certain effect. Differences in the expression between the sensitised and non-sensitised rats were tested with the Wilcoxon rank-sum test in R, and statistical significance was set to p-value <0.05. Log2FCs were visualised in R using the ggplot 2 package (Wickham, 2016).

### Ethics approval and consent to participate

The Ethical Committee of the School of Medicine at the University of Split (registry number 2181-198-03-04-18-004) and the Veterinary and Food Safety Office of the Ministry of Agriculture (registry number 525-10/0255-16-7) approved all animal experiments and protocols. Rat experiments were performed at the University of Split Animal Facility (permit number HR-POK-019) where specific pathogen-free animals were raised and housed in pairs, in plastic cages with sawdust and corn bedding. The animals were kept in a controlled environment: food and water ad libitum, temperature 22 ± 1°C, with a 12-h light/dark cycle. The animals were separated into individual cages and were food-deprived 24 h prior to the experiment.

European seabass experiments were undertaken in the experimental hatchery of the Institute of Oceanography and Fisheries, in concrete, 12-m^3^ flow-through tanks. Fish were purchased from a nearby farm and kept for an acclimation period of 30 days, fed their commercial diet, and exposed to a natural photoperiod. Water parameters (salinity and temperature) were measured every day by a probe. For details on the experiment, see Bušelić et al. (2018).

The datasets generated and analysed during the current study are available in the SRA repository, submitted under the BioProject PRJNA475982.

## Results

### RNA-seq and *de novo* assembly

Peripheral blood leukocytes (PBLs) obtained from the seabass and rat were stimulated by ACE for 1 and 12 h. Biological samples used for the RNA-seq were designated as FA1 (i.e., fish *Anisakis* 1 h, n = 3) and FA12 (i.e., fish *Anisakis* 12 h, n = 3) as well as and RA1 (i.e., rat *Anisakis* 1 h, n = 3) and RA12 (i.e., rat *Anisakis* 12 h, n = 3). For both species analysed, untreated PBLs (FC and RC, i.e., fish and rat control, n = 1/host) were included as a single biological sample. These controls were used to check the cell viability, and as an internal control of sequencing, but were excluded from downstream analyses (each represented a sample that included two technical replicates).

All material analysed with Bioanalyzer before RNA-seq showed RIN values higher than 8. For each host species, seven pairs of fastq files were produced. Reads obtained for sea bass PBLs were approximately 180 million, whereas reads for rat were approximately 140 million (**Table 2**). *De novo* assembly of PBLs from the sea bass yielded—following quality and expression filtering—112,232 contigs, whereas pooled reads of PBLs from rats yielded 217,917 contigs.

**Table 2:** RNA-seq data resulting from PBLs collected from *Dicentrarchus labrax* and *Rattus norvegicus* treated for 1 h and 12 h with *Anisakis* crude extract (also untreated PBL for each species and condition were included in the assembly and annotation analyses, whereas only treated samples were used for differential expression analyses).

	*Dicentrarchus labrax*	*Rattus norvegicus*
*De-novo assembly*		
Total pooled reads	183,624,017	191,848,162
Total Trinity genes	112,232	217,917
Total Trinity transcripts	146,252	272,379
Contig N50	2,328	1,790
Median contig length	333	403
*Annotation*		
Swissprot	19,158	26,358
Uniref	25,734	32,968
GO BP	7,510	18,570
GO MF	9,270	18,813
GO CC	8,361	19,749
*DEGs*		
up 1 h	1617	795
up 12 h	703	906

Info on reads obtained, de-novo assembly, annotation and DEGs with FDR < 0.05 are available.

### Differential gene expression analyses

**Figure 1:**
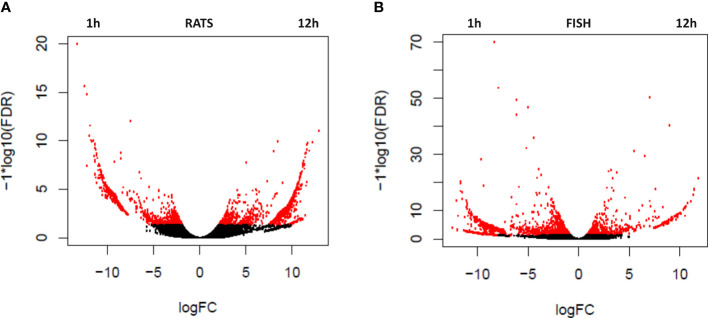
Volcano plots showing the relative expression levels of transcripts upregulated in the rat (*R. norvegicus*) **(A)** and fish (*D. labrax*) **(B)** peripheral blood leukocytes (PBLs) after 1 vs. 12 h of the treatment with *A. pegreffii* crude extract. The x-axis represents the log2 of the expression ratio for each transcript, and the y-axis represents the log10 of the p-value corrected for the false discovery rate. Red dots represent DEGs with logFDR < 0.05 (at least twofold difference in logCPM). Positive logFC values indicate transcripts enhanced after 12 h of treatment, whereas negative logFC values indicate transcripts enhanced after 1 h.

**Figure 2:**
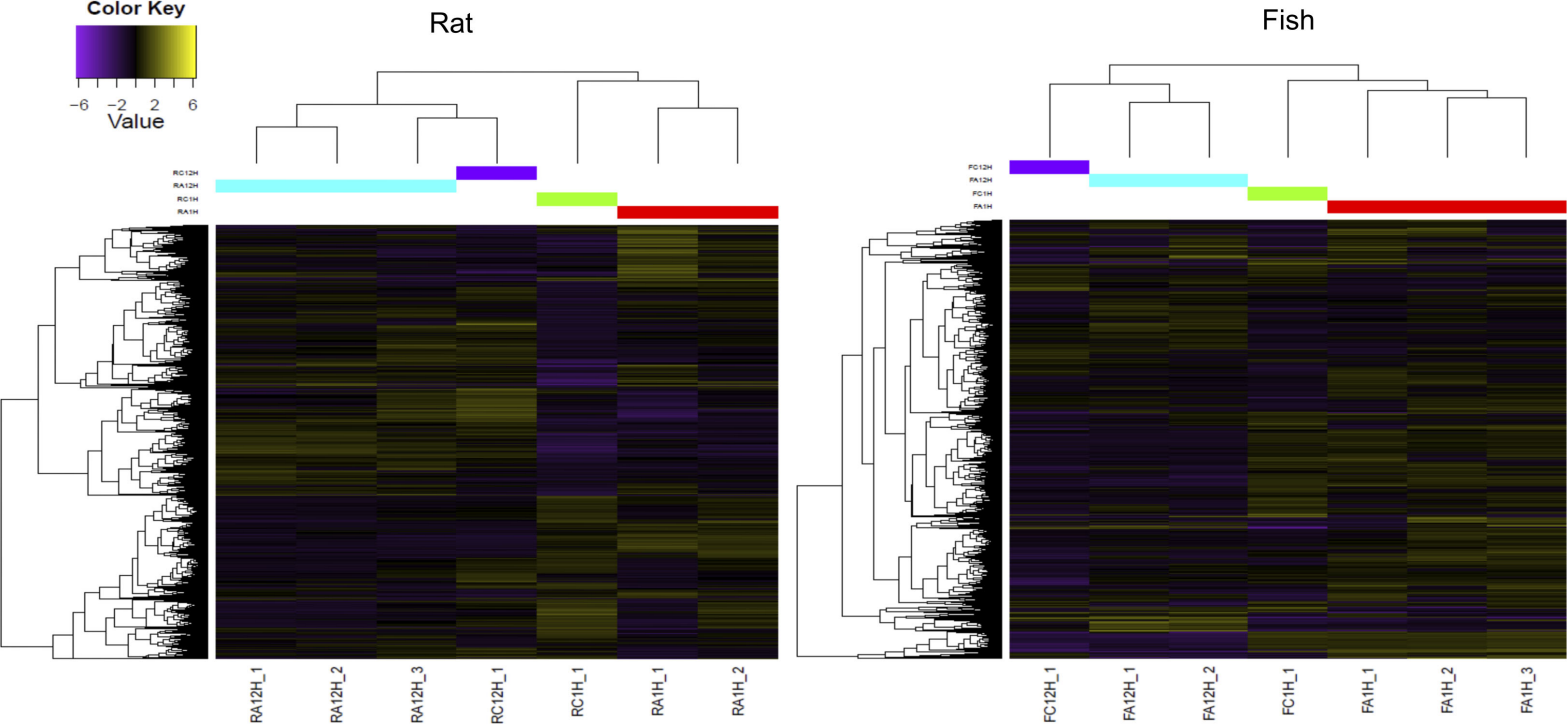
Heatmap showing highly significant differentially expressed genes (FDR <0.001 and logFC >2) in the rat (*R. norvegicus*) and fish (*D. labrax*) peripheral blood leukocytes (PBLs) after 1 vs. 12 h of treatment with *A. pegreffii* crude extract. Note that the unstimulated controls were excluded from differential expression analyses due to no replicate availability, but a single pooled sample is shown in the diagram for visual comparison. The colour scale represents the Log2 fold change (Log2FC). Different colours on the central upper bar indicate the different clusters to which each sample was related. Sample codes for the rat: RC1H_1—control 1 h, RC12H_1—control 12 h; RA1H_1, RA1H_2—biological replicas 1 h post-stimulation; RA12H_1, RA12H_2, RA12H_3—biological replicas 12 h post-stimulation; and for the fish: FC1H_1—control 1 h, FC12H_1—control 12 h; FA1H_1, FA1H_2, FA1H_3—biological replicas 1 h post-stimulation; FA12H_1, FA12H_2—biological replicas 12 h post-stimulation.

To evaluate the change in transcription in ACE-treated PBLs over time, differentially expressed genes were estimated comparing 1 and 12 h of treatment with ACE. Transcripts significantly upregulated (logFC >2 and FDR <0.05) are indicated in **Table 2** and **Figure 1**, as red dots in the volcano plots, whereas in the heatmap only highly significant DEGs have been included (logFC >2 and FDR <0.001) (**Figure 2**). A total of 2,320 differentially expressed transcripts were obtained comparing the two conditions in seabass PBLs and a total of 1701 in rat PBLs.

Annotation of the DEGs was performed on the highly significant subset of transcripts (FDR <0.001). Among 712 DEGs in the fish PBLs, 537 were annotated (75.4%) in the top 10 list according to logFC upregulated after 1 h of the treatment; we found transcripts encoding for zinc finger and BTB domain-containing protein 20, predicted histone-lysine N-methyltransferase 2C-like, N-terminal kinase-like protein SCY1-like protein 1, calcium-binding protein 39, thrombospondin-1, and un-annotated protein, and predicted egl nine homolog 1, transmembrane channel-like protein 6, and stromal interaction molecule 1-like. In the top 10 list of transcripts upregulated after 12 h of the treatment, we found motif of chemokines, interleukin-12 and 6, interferon-induced GTPases, and tumour necrosis factor receptor superfamily.

Among 493 DEGs in the rat PBLs, 472 were annotated (95.7%) in the top 10 list of transcripts upregulated after 1 h of the treatment; we found structural and gene-expression-related components as gamma-adducin, ribosomal protein kinase, metastasis-associated protein MTA3, and CCR4-NOT transcription complex, and transcripts with an essential role in the immune response as a T-cell surface glycoprotein CD8 precursor that interacts with TLRs and MHC molecules, as well as interferon-induced protein. The top 10 transcripts upregulated after 12 h also encompassed the structural elements, as well as regulators of translation and transcription. Detailed lists of the fish and rat PBLs DEGs are available as **Supplementary Material** (**Table S1**).

### Transcript annotation and enrichment analyses

Annotation of the rat and fish PBL transcriptomes was produced using the Annocript software (Musacchia et al., 2015). For the fish PBL transcripts, the repertoire of molecules identified consisted of 25,734 predicted peptides according to UniRef codes (UniProt Reference Clusters) inferred from 146,252 assembled transcripts, whereas for the rat PBL transcripts, 32,968 predicted peptides were obtained from 272,379 assembled transcripts. GO and Pfam enrichment analyses were performed on all significant DEGs (FDR <0.05).

In the fish PBLs treated for 1 h with *Anisakis* CE, DNA-dependent transcription and its regulation, together with intracellular signal transduction, cell adhesion, angiogenesis, neutrophil degranulation, and intracellular protein transport, were the most significantly enriched biological processes; DNA-RNA-ATP and metal ion binding were the most represented molecular function and cytoplasm, nucleus, membrane and extracellular vesicles the most represented cellular components. After 12 h in the fish PBLs, immune and inflammatory response and pro/anti apoptotic processes were also observed among the enriched biological processes, as well as chemokine, cytokine, and protein serine/threonine kinase activities among the enriched molecular functions. Cellular components were similar for the two time-points.

GO analyses in the rat PBLs after 1 h of the treatment showed pathways of transcription regulation, signal transduction and response to pathogens (against virus, bacteria, endocytosis process), and cellular response to interferon-beta among the most enriched biological processes. Binding activities (DNA, RNA, metal ions, and ATP) were among the enriched molecular function and cytoplasm, nucleus, cytosol, and extracellular vesicles among the most represented cellular components. After 12 h, DNA-dependent transcription was increased.

The pfam enrichment analyses confirmed the results from GO enrichment analyses: pfam enriched in 1- and 12-h subsets of DEGs in the fish PBLs represented by hundreds of units encompassed factors involved in immune response such as pfam13927 (immunoglobulin domain) and factors involved in cellular machinery such as pfam00076 (RNA recognition motif) and pfam00621 (Rho family members, GTPase activating protein). Similarly, pfam13637 (ankyrin repeats), pfam00017 (SH2-Src homology 2), pfam07654 (immunoglobulin C1-set domain), and pfam00059 (C-type lectin) were found enriched in 1-h-treated rat PBLs. Pfam07686 (immunoglobulin V-set domain), pfam00069 (protein kinase domain), and pfam00415 (regulatory of chromosome condensation) were found enriched after a 12-h treatment. Lists of enriched pfam for both hosts are available as **Supplementary Material** (**Tables S2**, **S3**).

### Cytokine elements in fish vs. rat early immune response kinetics

Fish PBLs 1 h post stimuli expressed C–X–C chemokine receptor type 3-2 (CXCR3), chemokine receptor 3 (CCR3), chemokine-like receptor 1 (CCR1), and interleukin-17 receptor E (IL-17RE). After 12 h post-stimuli, C–C motif chemokine 2-like (CCR2), C–C motif chemokine 20a.3 (CCL20), C–X–C motif chemokine 13 (CCL13), CC chemokine 1, chemokine-like receptor 1, interleukin 6, interleukin 8, interleukin 12, suppressor of cytokine signalling 1-like (SOCS1), TNF-alpha, and tumour necrosis factor receptor superfamily member 6B-like were also expressed.

Rat PBLs 1 h post stimuli expressed a predicted interleukin-6 receptor subunit alpha (IL-6R), pro-interleukin-16 (IL-16), and tumour necrosis factor alpha-induced protein 2, whereas the repertoire 12 h post stimuli included only cytokine receptor-like factor 3 (CRLF3).

### Ribosomal and nucleolar stress GO enriched in rat PBLs

We also searched for all the nucleolus- and ribosome-related transcripts in the rat PBLs to explore in more detail their differential expression between the two time points (**Table S4**). Ribosome-enriched transcripts counted 303 annotated proteins, the majority constituting 60S (N = 116) and 40S subunit (N = 52) proteins. Nucleolus-enriched transcripts accounted for 111 annotated proteins. However, only a single transcript in each category has been differentially upregulated: 60S ribosomal protein L13 (RPL13, 12 h post stimuli, 4.99 logFC) and inositol-pentakisphosphate 2-kinase (IPPK, 12 h post stimuli, 4.32 logFC).

### Target gene expression in rat spleen

**Figure 3:**
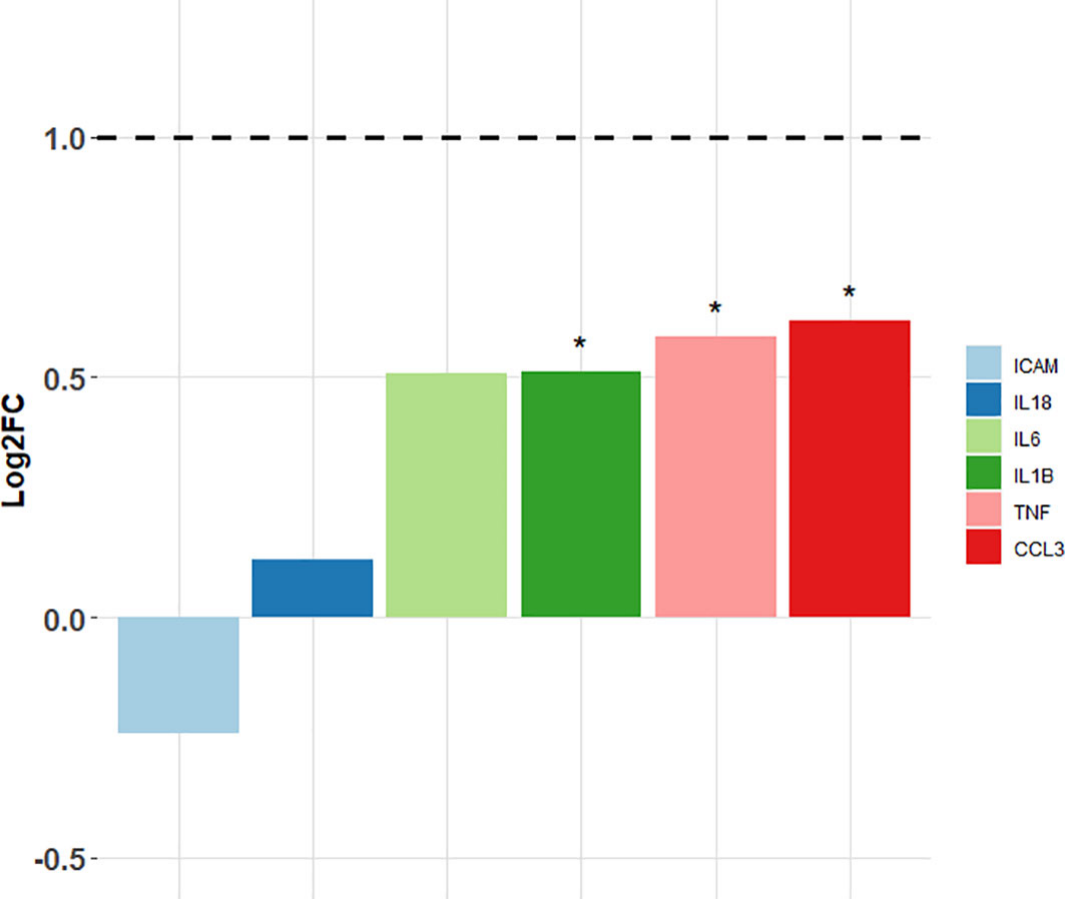
Log2FC values of the target genes measured in the sensitised rats vs. non-sensitised controls. Horizontal dashed line points to the fold change greater than 2 (i.e., log2FC ≥1) that was considered biologically significant. *Differentially expressed target genes (p < 0.05); ICAM—intra-cellular adhesion molecule-1; IL-18—interleukin-18; IL-6—interleukin-6; IL-1B—interleukin-1ß; TNF—tumour necrosis factor alpha; CCL3—chemokine (C-C motif) ligand 3.

Target gene expression was measured in spleens of the sensitised rats and compared with the unsensitised controls (**Figure 3**; **Table 3**). Out of six target genes, three were differentially expressed, namely, *Ccl3*, *Tnf-α*, and *Il1-ß*, in descending order of fold change. None of the selected target genes showed a biologically significant induction (LogFC ≥1). Interestingly, *Icam-1* was slightly downregulated in the sensitised rats, while *Il-18* showed virtually no change in the expression profile between the sensitised and unsensitised animals.

**Table 3:** Log2 transformed expression levels of the selected target genes for the sensitised rats and non-sensitised controls.

	Non-sensitised	Sensitised	Log 2FC	*p-value*
ICAM	7.857	7.614	-0.243	0.421
IL18	7.825	7.945	0.121	0.548
IL6	4.401	4.908	0.508	0.095
IL1B	**7.610**	**8.122**	**0.512**	**0.016**
TNF	**4.188**	**4.774**	**0.586**	**0.016**
CCL3	**5.295**	**5.911**	**0.616**	**0.008**

Values in bold indicate statistically significant differences between the sensitised rats and non-sensitised controls (p < 0.05).

## Discussion

The mRNA expressions observed herein serve as predictors of protein expressions (Guo et al., 2008). To better understand the transcripts’ functional roles in complex downstream pathways, we included corresponding protein products and their known functional roles.

### Fish vs. rat transcription 1 h post stimulation

Processes initiated in the fish PBLs 1 h post stimulation mainly involve explosive transcriptional activities starting up the TLR signalling, therefore still lacking abundant cytokine production and their downstream orchestration. The top expressed transcripts within 12–12.5 logFC expression levels are chiefly transcription factors, being turned off 12 h post stimuli.

The most expressed is the zinc finger and BTB domain-containing protein 20 (*Zbtb20*) that selectively suppresses the transcription of IκBα suppressor gene. This way, ZBTB20 promotes full activation of TLR signalling and TLR-triggered innate immune response (Liu et al., 2013), in particular the production of proinflammatory cytokines and type I interferon (IFN) in macrophages.

The second highly expressed transcript is *Kmt2c* (lysine methyltransferase 2C), which is a tumour suppressor mutated or deleted in the peripheral T-cell lymphoma (Watatani et al., 2019) and myelodysplastic syndrome (MDS) (Chen et al., 2021). KMT2C upregulation modifies histones during NK-cell activation in humans, helping the rapid shift in the expression of particular genes above the baseline during the target recognition process (Li et al., 2017). We can only speculate whether its effectors in the fish could be APC-type cells.

N-terminal kinase-like protein (SCY1) is another transcriptional regulator from the SCY1-like family of the kinase-like proteins. It orchestrates ER-Golgi intracellular trafficking, regulating the Golgi morphology and nuclear tRNA pathway (Pelletier, 2016). Interestingly, *Scy1* is highly upregulated in the CD4+ cells stimulated by tumour-derived antigens, resulting in induction of a regulatory phenotype of the T cell (Treg), which suggests its potential role in the tolerance mechanism during tumour development (Getnet et al., 2009).

The last highly upregulated transcript within the 12.0–12.5 logFC level is the calcium-binding protein 39 (*Cab39*). It forms a complex with a STE20 kinase, acting as a major upstream regulator of the AMP-activated protein kinase (AMPK) (Boudeau et al., 2003). In addition to having a role in metabolism, cell polarity, autophagy, and tumour progression, CAB39 is also recognised as an oncogenic factor (Xu et al., 2019). Interestingly, other calcium-binding proteins have a significant role in the neutrophil degranulation, but the role of CAB39 in the innate immunity has not been characterised yet.

Summing up the repertoire of the fish top-expressed transcripts, we infer that early stimulation of paratenic host PBLs expectedly manifests in TLR signalling start-up and abundant transcriptional activity targeting different subcellular compartments (i.e., nucleus and cytoplasm), all incentivising the proinflammatory pathway. However, it is intriguing to speculate whether SCY1 might contribute to skewing that response toward the tolerance as observed in the mice (Getnet et al., 2009), helping the larvae to eventually evade immunity and persist in a state of paratenesis for an indefinite time (Trumbić et al., 2021). The scenario of very early development of the *A. pegreffii* tolerance is further supported by a high expression of another multi-modular glycoprotein gene, thrombospondin-1 (*Tsp-1*). *Tsp-1* expression mediated by TGF-β2-expressing APCs facilitates activation of the latent transforming growth factor TGF-β in an integrin-independent way. Namely, whereas integrin-mediated activation of latent TGF-β1 is essential for the induction of Treg by APCs, the TGF-β2 isoform lacks an integrin-binding RGD sequence and therefore undergoes integrin-independent activation *via* TSP-1. Expression of TGF-β2 by APCs stimulates FOXP^3+^ Treg cells both *in vitro* and *in vivo* (Mir et al., 2015).

In the rat, the accidental host model, processes activated by *A. pegreffii* encompass strong T-cell activation, guided by highly expressed gamma-adducin isoform 1 (*Add1*). By assembling the spectrin–actin network, ADD1 physically supports the plasma membrane, also mediating signal transduction in different cellular pathways (Kiang and Leung, 2018). The role of ADD1 in the CD4+ and CD8+ T-cell activation and co-stimulation of CD28 has been recently evidenced (Thauland et al., 2019), suggesting that *A. pegreffii* in the naïve rat could stimulate differentiation of both killer and helper T cells. Differentiation toward the T killer cells is further supported by a high expression of the T-cell surface glycoprotein CD8 alpha chain precursor gene, which primarily serves as a co-receptor for the MHC class I molecule:peptide complex on cytotoxic cells. It seems that while at the same time-point fish PBLs manifest the initiation of TLR pathways, rat PBLs already undergo T-cell differentiation.

Another highly expressed transcript is the ribosomal protein S6 kinase alpha-3 (*Rps6ka3*) that engages downstream of ERK (MAPK1/ERK2 and MAPK3/ERK1) signalling, activating transcription factors for cell proliferation, survival, and differentiation through mTOR signalling modulation and suppression of the pro-apoptotic factors. RPS6KA3 is also involved in macropinocytosis of lipoproteins mediated by bacterial LPS-activated TLR4 of the dendritic cells. In evolutionary well-established host–parasite systems, such as visceral leishmaniasis (VL) caused by *Leishmania* spp., TLR4 detects glycosphingophospholipid derived from *L. donovani* and the proteoglycolipid complex (P8GLC) from *L. pifanoi*, enhancing the production of proinflammatory cytokines. To counteract this, *L. donovani* alters TLR4 signalling to establish its infection. However, TLR4 signalling also hampers the chronic immune response during VL through TLR4’s activation of IRF1 and consecutive upregulation of IFN-β, which in turn acts directly on the Th1 cells to limit the production of IFN-γ (Sacramento et al., 2020). If in the rat-*Anisakis* model RPS6KA3 indeed stimulates TRL4 signalling, we speculate that the signalling would be directed toward upscaling of the proinflammatory reaction, rather than skewing of the pathway to favour parasite infection. This is supported also by upregulation of the unconventional myosin-Ig (*Myo1g*) that engages in the T-cell migration toward rare antigens (Gérard et al., 2014). MYO1G is a plasma membrane-associated class I myosin also expressed in the B cells that regulates the Fc-gamma receptor signalling pathway during endocytosis of external particulate material (Maravillas-Montero et al., 2014). It is equally feasible that 1 h post stimulation of the rat PBLs, *Myo1g* expresses in both T- and B-cell linages.

The rest of the top 10 highly expressed transcripts (logFC >12) contribute to cell proliferation through regulation of mRNA and protein budding (genes CCR4-NOT transcription complex subunit 6, eukaryotic translation initiation factor 2 subunit 2, scaffold attachment factor B2). The exception is the interferon-induced protein gene with tetratricopeptide repeats 3 (*Ifit3*) that has multiple roles, such as the inhibition of both cellular and viral processes, cell migration, proliferation, signalling, and viral replication (Zhou et al., 2013), but also negative regulation of apoptosis and response to PAMPs (Pidugu et al., 2019). While it triggers nuclear antiviral gene transcription, IFIT3 also induces anti-proliferative activity *via* the upregulation of cell-cycle negative regulators CDKN1A/p21 and CDKN1B/p27. Additionally, it also negatively regulates the apoptotic effects of IFIT2. It is likely that *A. pegreffii* PAMPs trigger *Ifit3* in rat PBLs and that the protein consequently counteracts the immune cell proliferation through p21 and p27 and/or blocks their apoptosis.

### Fish vs. rat transcription 12 h post stimulation

Interestingly, the top expressed cytokine in fish PBL is the interleukin 12 subunit b gene (*Il-12b*, *p40*). In the early response as herein, IL-12b stimulates the production of IFN-gamma from the NK and T cells, helping in activation of phagocytic cells and supporting Th1-cell differentiation (Trinchieri, 1995). Although primarily a proinflammatory cytokine, IL-12b also associates with the p19 subunit of interleukin-23, which together with IL-6 and IL-1 is able to drive naïve CD4+ T cells to become Th17 cells independent of TGF-β1 signalling (Ghoreschi et al., 2010). These Th17 cells are then able to acquire Th1 or Th2 phenotypes at later stages of the cell differentiation. It is interesting to note that *Tnf-*α and *Il-6*, but not *Il-1β*, are upregulated 12 h post stimuli, which suggests that at this point it is still not clear which direction the T-cell differentiation will assume. However, at 12 h post stimuli there is the first evidence of B-cell activation, through 11.4 logFC expression of the insulin receptor substrate 2-B gene (*Irs2*). Its product, a large cytoplasmic docking protein, is recruited to IL-4Rα by its first cytoplasmic tyrosine residue (Keegan et al., 1994). The binding consequently mediates proliferative and antiapoptotic signalling through the IL-4R, regulating IgE and IgG1 production by B cells and lymphokine-producing phenotype of CD4+ Th cells (Kelly-Welch et al., 2004). Herein, expressed *Irs2* could be an important enhancer of anti-apoptosis in lymphocytes.

Olfactomedin-4 (*Olfm4*) is a glycoprotein gene studied mostly in relation to the digestive system pathologies; in gastrointestinal malignancies, it has a role as an antiapoptotic factor, in *Helicobacter pylori* infection it downregulates innate immunity through the negative feedback on the NF-κB pathway, whereas in the inflammatory bowel diseases it affects the anti-inflammatory function (Wang et al., 2018). Human and mouse neutrophils express OLFM4 phenotype early during cell differentiation and in relation to sepsis, but since its overexpression is correlated with worse outcome, it is suggested that these neutrophils or the OLFM4 secreted may be pathogenic (Alder et al., 2019). Interestingly, when transcriptomes of the Japanese (*Anguilla japonica*) and the European eel (*Anguilla anguilla*) (Bracamonte et al., 2019) infected by the nematode *Anguillicola crassus* were compared, *Olfm4* was significantly downregulated in the Japanese eel, which is the native host for this parasite, comparable with our fish model (native host for *A. pegreffii*). Bracamonte et al. (2019) observed a low number of differentially expressed immune-related genes, especially in the native host, suggesting that the parasite exerts a minor impact on the immune response during the early stages of infection (i.e., 3 days) in the native host, but not newly acquired host. Although we cannot draw a strong comparison with the eel model, as herein we studied two phylogenetically distinct hosts, implication of OLFM4 in regulation of inflammation during the infection with an evolutionary-established parasite deserves further research.

The next highly expressed transcript 12 h post stimuli in the fish is the voltage-dependent calcium channel subunit alpha-2/delta-1a, playing an important role in excitation–contraction coupling. This proves the onset of T-cell differentiation, as the resting lymphocytes maintain a low concentration of Ca^2+^, whereas their engagement of antigen receptors induces calcium influx from the extracellular space by several routes (Vig and Kinet, 2009). A very important master kinase expressed at this point is 3-phosphoinositide-dependent protein kinase 1 gene (*Pdpk1*), whose product phosphorylates and activates a subgroup of the AGC family of the protein kinases. In relation to immune response, it regulates Ca^2+^ entry and Ca^2+^-activated K^+^ channels of mast cells, activates the NF-κB pathway, regulates pre-T-cell expression of key nutrient receptors mediating Notch-induced cell growth and proliferative responses, and finally inhibits the TLR-mediated NF-κB activation in macrophages.

Lastly, a high expression of the FK506-binding protein 12 gene might contribute to pro-inflammatory silencing, as the protein has a key role in immunosuppressant-mediated immuno-suppression in the T cells (Xu et al., 2002).

In the rat PBLs 12 h post stimuli, a top regulated effector transcript, MAP kinase-interacting serine/threonine-protein kinase 2 (*Mknk2*), is found overexpressed (12.8 logFC). Its product MKNK2 binds to phosphorylated eukaryotic translation initiation factor 4E (eIF4E), leading to cell proliferation or differentiation through the ERK-MAPK pathway (Scheper et al., 2001), which is essential for signal transduction in T cells (Rincón et al., 2001). The MAPK/Erk is initiated downstream of the T-cell receptor activation, being indispensable for primordial T cells to proliferate, grow, exert their effector functions, and survive, as well as differentiation of both the IFN-γ-producing Th1 cells (Tripathi et al., 2012) and IL-4/13-producing Th2 cells (Chang et al., 2012). This suggests that 12 h post stimuli the fate of activated T cells is still vague, similar to the fish model.

Other highly expressed transcripts have a role in the coordination of generic regulatory processes, such as the little elongation complex subunit 1 gene (*Ice1*), required by RNA polymerase II and III to regulate small nuclear RNA (snRNA) gene transcription (Hu et al., 2013).

Interestingly, a group of transcripts important for cell motility are highly expressed in the rat PBLs 12 h post stimuli. One is plectin isoform 1a gene (*Plec1*), with an essential role in the motility of T and dendritic cells. The second is the cutaneous T-cell lymphoma-associated antigen 5 gene (*ctage5*), a tumour antigen on cutaneous T-cell lymphoma (CTCL) whose function has been related to protein, lipoprotein, insulin, and collagen secretion, transport of cellular (non-secretory) components of neurones, brain development, and expansion of plasma membrane (Zhang et al., 2018). The role of cTAGE5 in the immune response has not been characterised, except for observed upregulation in the macrophage cytosolic proteomes stimulated by *Herpes simplex* virus-1 (Miettinen, 2014). *Ctage5* overexpression in the rat PBLs 12 h post stimuli is significant (11.7 logFC), and we can speculate that it might have a role in leukocytes’ mobility or protein secretion. Lastly, the microtubule-actin cross-linking factor 1 gene is important in the cross-linking and stabilisation of actin and microtubules, engaged in the focal adhesion size and cell migration, whereas utrophin present in cell membranes binds actin filaments.

The only highly expressed (11.5 logFC) transcriptional factor at this time-point is the AT-hook-containing transcription factor, which regulates CD40 and CD40 ligand expression. It is expressed by B and T lymphocytes, NK cell, and dendritic cells, in particular at the stage of the B-cell receptor and ligand interactions, being crucial for the B-lymphocyte maturation (Siddiqa et al., 2001).

### Cytokine genes in fish vs. rat early immune response kinetics

The interactive cross-talk mediated by cytokine genes in fish at both time points suggests early recruitment of effector immune cells to the site of inflammation, supported by a simultaneous high expression of transcriptional factors initiating TLR signalling and T-cell activation. Cytokine gene expression is more intense in the fish, where we expected a more tolerant signalling environment.

The fish repertoire at 1 h post stimuli indicates the cells’ potential to differentiate toward all possible outcomes, i.e., Th1, Th2, and Th17 cells. For instance, CXCR3 that is expressed mainly on activated Th1 and NK cells (Qin et al., 1998) is targeted by ligands that concomitantly block the migration of Th2 cells in response to CCR3 ligands, the latter being the main receptor on the differentiated Th2 cells and simultaneously expressed herein. IL-17RE in addition binds the proinflammatory cytokine IL-17B, being afterward highly expressed in Th17 cells (Chang et al., 2011). At 12 h post stimuli, there is a strong chemotactic response and mobilisation of the intracellular calcium ions (*Ccr2*), interleukin-17 signal activation, and chemoattraction of Th17 cells (*Ccr20*) and B cells (*Ccl13*), and a general proinflammatory milieu (*Il-1*, *Il-6*, *Il-8*, *Il-12*, *Tnf-α*). The latter seems to be balanced or attenuated by *Socs1* expression that, in a negative feedback loop, attenuates cytokine signalling.

Unexpectedly, a scarce cytokine transcript environment was observed in the rat at both stimulation time points, evidencing a proinflammatory character (*Il6r*, *Tnf-α*) directed toward chemoattraction of the CD4+ cell (*Il16*). The only related element retrieved from the enrichment analysis was the IL-4 receptor gene, involved in the regulation of IgE production. The only cytokine expressed after 12 h is *Crlf13* that negatively regulates cell-cycle progression, which potentially may indicate the balancing of the proinflammatory cell differentiation (Yang et al., 2009).

Majority of these ligands and receptors in both host models, in addition to their chemoattractant properties, also stimulate angiogenesis.

Interestingly, weak cytokine induction was also recorded in spleens of the sensitised rats. Although *Ccl3*, *Tnf-α*, and *Il-1ß* were differentially expressed, none of these genes showed biologically significant induction, regarded as logFC ≥1. IL-1ß is a canonical proinflammatory cytokine and a major endogenous pyrogen, which activates neutrophils and macrophages. In addition to this, IL-1ß also stimulates the secretion of the other two canonical proinflammatory cytokines, TNF and IL-6 (Netea et al., 2010). While *Tnf-α* was differentially expressed, we evidenced no differential expression of *Il-6*. Contrary to these results, Hrabar et al. (2019) observed a very strong induction of *Il-6* in stomach and visceral adipose tissue of experimentally infected rats, among other mediators of inflammation. Furthermore, both IL-1ß and IL-6 can induce a bias toward the Th17 phenotype (Netea et al., 2010; Scheller et al., 2011) and accordingly Bušelić et al. (2018) found an upregulation of the IL-17 signalling pathway in the transcriptome of muscle and stomach of the experimentally infected rats. Furthermore, rats infected with live *Anisakis* larvae had elevated levels of serum IL-17 compared with those inoculated with CE, whereas neither of the group had elevated IL-6 levels (Abdel-Ghaffar et al., 2015). CCL3 is a chemokine, also with pyrogenic properties, primarily acting as a neutrophil chemoattractant (Davatelis et al., 1989), and its strong upregulation was also found in the stomach, muscle, and visceral adipose tissue of the experimentally infected rats (Bušelić et al., 2018; Hrabar et al., 2019). In *in vitro* studies with human monocyte-derived dendritic cells, both live *Anisakis* larvae and CE stimulated the secretion of CCL3, together with other proinflammatory cytokines, such as IL-6 and IL-1α (Napoletano et al., 2018). Taken together, these findings would suggest a differential role of IL-17 and potential bias toward Th17 response in immunity to *Anisakis*. This seems to be dependent on the mode of infection, with potentially live *Anisakis* larvae inducing this bias.

### Ribosomal and nucleolar stress GO enriched in rat PBLs

Increased ribosynthesis is a cell strategy to retrieve homeostasis (Sengupta et al., 2010) during stress conditions that affect the cell cycle and intracellular energy status, recognised also as essential for efficient immune response signalling and orchestrating (Wan et al., 2007; Mukhopadhyay et al., 2009; Gao and Hardwidge, 2011). In consequence, a higher demand for ribosynthesis reflects on the nucleolar architecture and production, the latter being responsible for transcription and processing of precursor ribosomal RNA (pre-rRNA) and assembly of precursors of the small and large ribosome subunits. Therefore, concomitant ribosomal and nucleolar perturbations are related to changes in functional state of the cell, especially during immune response (Tan et al., 2017), which was the reason to scrutinise these targets herein. Our previous research of rat transcriptomes evidenced that *Anisakis*-infected stomach tissues undergo ribosomal stress in the early infection stage (Bušelić et al., 2018). Its KEGG 03010 ribosome pathway was characterised by the highest q value and consistent, but not extensive, gene upregulation. However, we have found no common elements between highly expressed ribosomal DEGs in the previous studies’ PBLs (*Rsl27*, *Rlp22*) and this study’s PBLs (*Rlp13*), probably due to the different type of tissues, sampling times (pooled 6–32 h post infection vs. 12 h post stimuli), and type of the stimulus (live larvae vs. ACE). It is interesting to note that a recent study identified RPL13 having a role in the induction and activation of the promoters of the nuclear factor-κB (NF-κB) and interferon-β (IFN-β) genes, and the expression and protein secretion of the antiviral factor IFN-β and proinflammatory cytokine interleukin-6 in the course of foot-and-mouth disease viral response (Guan et al., 2021).

This supports our observation of *Rpl13* being involved in the course of immune response to parasitic antigen stimulation. Nucleolar stress has been visualised in the *Anisakis*-stimulated rat peripheral blood mononuclear cells 12 h post stimuli in the form of nucleolar segregation. The alteration was characterised by architectural changes of nucleolar components; enlargement of the dense fibrillar component (DFC), development of multiple, large and dense, mulberry-like granular components (GC), and speckling of nucleophosmin. Genes recognised as markers for these changes are nucleolar protein 58 (*Nop58*), expressed in DFC and required for 60S biogenesis, and nucleophosmin (*Npm1*), expressed in GC and recognised as a multifunctional cellular chaperon, both expressed in the rat PBLs at both sampling time points. This observation suggests that alteration of nucleolar function in PBLs is mediated by *Anisakis* antigens as early as 1 h post stimuli. However, the only nucleolus-related DEG upregulated 12 h post stimuli was inositol-pentakisphosphate 2-kinase gene (*Ippk*). Its product, IPPK enzyme, is concentrated in the discrete cellular foci within the nucleolus and catalyses the phosphorylation of inositol phosphates (*InsPs*). *InsPs* are soluble signalling molecules with multiple cellular roles (Verbsky and Majerus, 2005; González et al., 2010; Shears, 2010), but most importantly in the T-cell activation (Guse and Emmrich, 1991) and conversely in B-cell antigen receptor signalling in T-cell-independent humoral immunity (Kim et al., 2019). Its increased activation 12 h post stimuli observed herein is in line with the general upregulation of activities related to T-cell differentiation, although elucidation of its exact role needs more elaborated experimental design.

## Conclusions

Rat and fish, the two evolutionary distinct host models for *A. pegreffii* interaction, employ qualitatively different transcript cascades achieving the same effect at an early time scale of the innate immunity response. Fish 1-h-stimulated PBLs respond by explosive transcriptional activities that ignite the TLR signalling, eventually followed by a pro-inflammatory cascade triggered by Il-12b 12 h post-stimulation. Consequently, this helps in activation of phagocytic cells and supports Th1-cell differentiation. In contrast, based on expressed transcripts, rat 1-h-stimulated PBLs already show a strong T-cell activation, although T cells’ fate in terms of differentiation toward Th1 vs. Th2 is still vague 12 h post-stimulation. Whether implication of a latter immunity element alone, or in combination with other hosts’ intrinsic conditions (temperature, microbiome composition), in particular the % of immune cell in PBL fraction (Esteban et al., 2000) contributes to death of L3 in the terrestrial host needs further investigation.

## Data availability statement

The datasets presented in this study can be found in online repositories. The names of the repository/repositories and accession number(s) can be found in the article/**Supplementary Material**.

## Ethics statement

The animal study was reviewed and approved by The Ethical Committee of the School of Medicine at the University of Split (registry number 2181-198-03-04-18-004) and the Veterinary and Food Safety Office of the Ministry of Agriculture (registry number 525-10/0255-16-7).

## Author contributions

Conceptualisation, IM and SC; methodology, IM, SC, MP, and JH; software, MS and SC; validation, IM, SD, and SC; formal analysis, MS, SC, MP, and JH; investigation, IM and SD; resources, IM; data curation, IM; writing—original draft preparation, IM, MS, SC, and JH; writing—review and editing, IM, SC, JH, and SD; visualisation, SC and JH; supervision, IM and SD; project administration, IM; funding acquisition, IM. All authors have read and agreed to the published version of the manuscript.

## Supplementary material

Supplementary Table 1 List of differentially expressed transcripts in the peripheral blood leukocytes (PBLs) of fish (*Dicentrarchus labrax*) and rat (*Rattus norvegicus*) stimulated by *Anisakis pegreffii* crude extract 1 and 12 h post-stimulation. Information about transcripts ID, logFC, logCPM, p-value, FDR, annotation, PFAM code and hyperlink to NCBI repository are available.

Supplementary Table 2 List of enriched pfam of upregulated genes in the peripheral blood leukocytes (PBLs) of fish (*D. labrax*) stimulated by *A. pegreffii* crude extract 1 and 12 h post-stimulation.

Supplementary Table 3 List of enriched pfam of upregulated genes in the peripheral blood leukocytes (PBLs) of rat (*R. norvegicus*) stimulated by *A. pegreffii* crude extract 1 and 12 h post-stimulation.

Supplementary Table 4 List of ribosome- and nucleolar stress-enriched transcripts in the rat (*R. norvegicus*) peripheral blood leukocytes (PBLs) stimulated by *A. pegreffii* crude extract 1 and 12 h post-stimulation. Note that only 60S ribosomal protein L13 (RPL13, 12 h post stimuli, 4.99 logFC) and inositol-pentakisphosphate 2-kinase (IPPK, 12 h post stimuli, 4.32 logFC) have been differentially expressed between 1 and 12 h post-stimulation.
